# Supplementary material for: Reproducibility of strength performance and strength-endurance profiles: A test-retest study
Source: PLoS One. 2022 May 5;17(5):e0268074. doi: 10.1371/journal.pone.0268074 (PMC9070879; doi:10.1371/journal.pone.0268074)
Supplement: S1 Appendix — This file contains detailed information on priors and models. (PDF) [file pone.0268074.s001.pdf]

Supporting Information 1: Modeling details

1. Fundamental Bayesian workflow

Each analysis followed the same Bayesian workflow:

- First, a model was defined based on the associated research question and underlying assumptions (e.g. normal distribution of residuals).
- Second, priors were defined for each model parameter, with the main goals of making a-priori implausible values unlikely and limiting the parameter space to logical boundaries, e.g. positive values for scale parameters (standard deviations). For this purpose, weakly informative priors were defined based on expert information. In a few exceptions, priors were based on the results of a preceding pilot study to facilitate chain convergence during sampling. A prior sensitivity analysis was conducted by introducing a set of three competing priors with larger scales, following a similar approach to Deapoli and colleagues [1]. A prior was considered appropriate (i.e. truly weakly informative) when the posterior overlap with the competing priors was at least 90%.
- Third, posterior distributions of model parameters were estimated by using the Hamiltonian Monte Carlo (HMC) algorithm of the probabilistic programming language Stan [2]. Four chains sampled from random initial values for 4000 iterations, respectively, the first 2000 iterations of each chain being discarded as warm-up.
- Fourth, sampling diagnostics (R-hat, number of divergent transitions, effective sample size, and traceplots) were evaluated to ensure chain convergence and reliability of the parameter estimation process. In particular, the following criteria had to be fulfilled to assume reliable posterior estimates: (1) R-hat < 1.05, (2) no divergent transitions, (3) effective sample size > 1000, and (4) visual convergence of all chains. When necessary, sampling specifications (HMC acceptance rate, number of iterations) or model specifications (parameterization) were adapted, as suggested by the software developer.

2. Reproducibility of performance measures

2.1. Model

$P_{ij} \sim Normal(\mu + s_i + \Delta t D_j,\ {\sigma_e}^2)$

$s_i \sim Normal(0,\ {\sigma_s}^2)$

, where $i = \{1, \dots, n_{sub}\}$ and $j = \{1, 2\}$

2.2. Priors

A weakly informative zero-centered normal prior was selected for the fixed effect of time (Δt) to represent the uncertainty about whether the test protocol allowed for systematic positive (learning effect) or negative (fatigue-induced) changes in performance between trials. For σ_e and σ_s, weakly informative Half-Cauchy priors were defined. For the fixed effect of performance at T1 (μ), a weakly informative prior was defined specifically for each performance measure: The location parameters were based on published norm values for the 1-RM in the bench press [3] and population-average guidelines for the RTF at 90%, 80%, and 70% of the 1-RM [4]. The scale parameters were chosen conservatively to minimize the amount of information drawn from the prior.

$\Delta t \sim Normal(0, 10)$[1]

$\sigma_e \sim Cauchy(0, 10),\ \sigma_e \in [0, \infty]$

$\sigma_s \sim Cauchy(0, 10),\ \sigma_s \in [0, \infty]$

For (P = 1-RM): $\mu \sim Normal(50, 50),\ \mu \in [0, \infty]$

For (P = RTF at 90% 1-RM): $\mu \sim Normal(4, 2),\ \mu \in [0, \infty]$

For (P = RTF at 80% 1-RM): $\mu \sim Normal(8, 4),\ \mu \in [0, \infty]$

For (P = RTF at 70% 1-RM): $\mu \sim Normal(11, 6),\ \mu \in [0, \infty]$

[1] If the parameter space is not explicitly defined, it is considered unbounded, e.g. $\Delta t \in [-\infty, \infty]$.

3. Reproducibility of strength-endurence models

3.1. Models

$$Lin\text{: } load_{ij} \sim Normal((\boldsymbol{a_i} + \Delta\boldsymbol{a_i}D_j) + (\boldsymbol{b_i} + \Delta\boldsymbol{b_i}D_j)RTF, \sigma^2)$$

$$Ex2\text{: } load_{ij} \sim Normal((\boldsymbol{a_i} + \Delta\boldsymbol{a_i}D_j)e^{\left((\boldsymbol{b_i}+\Delta\boldsymbol{b_i}D_j)RTF\right)}, \sigma^2)$$

$$Ex3\text{: } load_{ij} \sim Normal((\boldsymbol{c_i} + \Delta\boldsymbol{c_i}D_j) + (\boldsymbol{a_i} + \Delta\boldsymbol{a_i}D_j)e^{\left((\boldsymbol{b_i}+\Delta\boldsymbol{b_i}D_j)RTF\right)}, \sigma^2)$$

$$Crit\text{: } load_{ij} \sim Normal((\boldsymbol{L'_i} + \Delta\boldsymbol{L'_i}D_j) \;/\; (RTF - (\boldsymbol{k_i} + \Delta\boldsymbol{k_i}D_j)) + (\boldsymbol{CL_i} + \Delta\boldsymbol{CL_i}D_j), \sigma^2)$$

, where $i = \{1, \ldots, n_{sub}\}$ and $j = \{1, 2\}$

The hierarchical structure for the models using 2 parameters (*Lin* and *Ex2*) was modeled as:

$$\begin{pmatrix}\boldsymbol{a_i}\\ \boldsymbol{b_i}\end{pmatrix} \sim Normal\left(\begin{pmatrix}\mu_a\\ \mu_b\end{pmatrix}, \boldsymbol{\Sigma_{ab}}\right)$$

$$\boldsymbol{\Sigma_{ab}} = \begin{pmatrix}\tau_a^2 & \rho_{ab}\tau_a\tau_b\\ \rho_{ab}\tau_a\tau_b & \tau_b^2\end{pmatrix} = \begin{pmatrix}\tau_a & 0\\ 0 & \tau_b\end{pmatrix}\boldsymbol{L_u}\boldsymbol{L_u^T}\begin{pmatrix}\tau_a & 0\\ 0 & \tau_b\end{pmatrix}$$

$$\begin{pmatrix}\Delta\boldsymbol{a_i}\\ \Delta\boldsymbol{b_i}\end{pmatrix} \sim Normal\left(\begin{pmatrix}\mu_{\Delta a}\\ \mu_{\Delta b}\end{pmatrix}, \boldsymbol{\Sigma_{\Delta a\Delta b}}\right)$$

$$\boldsymbol{\Sigma_{\Delta a\Delta b}} = \begin{pmatrix}\tau_{\Delta a}^2 & \rho_{\Delta a\Delta b}\tau_{\Delta a}\tau_{\Delta b}\\ \rho_{\Delta a\Delta b}\tau_{\Delta a}\tau_{\Delta b} & \tau_{\Delta b}^2\end{pmatrix} = \begin{pmatrix}\tau_{\Delta a} & 0\\ 0 & \tau_{\Delta b}\end{pmatrix}\boldsymbol{L_v}\boldsymbol{L_v^T}\begin{pmatrix}\tau_{\Delta a} & 0\\ 0 & \tau_{\Delta b}\end{pmatrix}$$

The hierarchical structure for the models using 3 parameters (*Ex3* and *Crit*) was modeled as:

$$\begin{pmatrix}\boldsymbol{a_i}\\ \boldsymbol{b_i}\\ \boldsymbol{c_i}\end{pmatrix} \sim Normal\left(\begin{pmatrix}\mu_a\\ \mu_b\\ \mu_c\end{pmatrix}, \boldsymbol{\Sigma_{abc}}\right)$$

$$\boldsymbol{\Sigma_{abc}} = \begin{pmatrix}\tau_a^2 & \rho_{ab}\tau_a\tau_b & \rho_{ac}\tau_a\tau_c\\ \rho_{ab}\tau_a\tau_b & \tau_b^2 & \rho_{bc}\tau_b\tau_c\\ \rho_{ac}\tau_a\tau_c & \rho_{bc}\tau_b\tau_c & \tau_c^2\end{pmatrix}$$

$$= \begin{pmatrix}\tau_a & 0 & 0\\ 0 & \tau_b & 0\\ 0 & 0 & \tau_c\end{pmatrix}\boldsymbol{L_u}\boldsymbol{L_u^T}\begin{pmatrix}\tau_a & 0 & 0\\ 0 & \tau_b & 0\\ 0 & 0 & \tau_c\end{pmatrix}$$

$$\begin{pmatrix}\Delta\boldsymbol{a_i}\\ \Delta\boldsymbol{b_i}\\ \Delta\boldsymbol{c_i}\end{pmatrix} \sim Normal\left(\begin{pmatrix}\mu_{\Delta a}\\ \mu_{\Delta b}\\ \mu_{\Delta c}\end{pmatrix}, \boldsymbol{\Sigma_{\Delta a\Delta b\Delta c}}\right)$$

$$\boldsymbol{\Sigma}_{\boldsymbol{\Delta a \Delta b \Delta c}} = \begin{pmatrix} \tau_{\Delta a}^2 & \rho_{\Delta a \Delta b}\tau_{\Delta a}\tau_{\Delta b} & \rho_{\Delta a \Delta c}\tau_{\Delta a}\tau_{\Delta c} \\ \rho_{\Delta a \Delta b}\tau_{\Delta a}\tau_{\Delta b} & \tau_{\Delta b}^2 & \rho_{\Delta b \Delta c}\tau_{\Delta b}\tau_{\Delta c} \\ \rho_{\Delta a \Delta c}\tau_{\Delta a}\tau_{\Delta c} & \rho_{\Delta b \Delta c}\tau_{\Delta b}\tau_{\Delta c} & \tau_{\Delta c}^2 \end{pmatrix}$$

$$= \begin{pmatrix} \tau_{\Delta a} & 0 & 0 \\ 0 & \tau_{\Delta b} & 0 \\ 0 & 0 & \tau_{\Delta c} \end{pmatrix} \boldsymbol{L_v L_v^T} \begin{pmatrix} \tau_{\Delta a} & 0 & 0 \\ 0 & \tau_{\Delta b} & 0 \\ 0 & 0 & \tau_{\Delta c} \end{pmatrix}$$

, where $L_u L_u^T$ and $L_v L_v^T$ describe the Cholesky decomposition of the respective correlation matrix.

The following equations account for different parameter labels in *Crit*. Importantly, the two parameters L’ and ΔL’ were reparameterized to facilitate sampling of the HMC algorithm:

$L' = 100 \cdot a, L' \in [0, \infty]$

$\Delta L' = 100 \cdot \Delta a$

$k = b, k \in [-\infty, 0]$

$\Delta k = \Delta b$

$CL = c$

$\Delta CL = \Delta c$

The selected boundaries for *L’* and *k* were chosen as suggested by Morton and colleagues [5] to achieve the characteristic hyperbolic shape of the function and allow for a y-intercept. However, we decided not to limit *CL* to positive values as suggested by the authors. Morton and colleagues reported a concentration of *CL* estimates at 0 for 12 out of 16 subjects when defining a [0, ∞] boundary [5]. Since this could be interpreted as an improper parameter truncation, we advocate that *CL* should be free to vary across positive and negative values.

3.2. Priors

For change effects, weakly informative zero-centered normal priors were defined to represent the uncertainty about the direction of parameter changes. Weakly informative Half-Cauchy priors were applied for scale parameters (τ and σ). For the group-level parameters at T1, priors were defined by moment-matching the posterior distributions of a pilot study that was conducted on a different sample of eight subjects, in order to facilitate chain convergence during HMC sampling. These subjects were not included in the main analysis because they did not undergo a retest, hence only providing data for T1. Moreover, subjects of the pilot study

performed the bench press without using safety pins as delimiter for the eccentric phase of the movement, but instead performed a standard touch-and-go bench press. To reduce the information provided by these "pilot-informed" priors and therefore fulfill the previously described prerequisite for truly weakly informative priors, the scale of the moment-matched posterior distribution of the pilot study was multiplied by the factor 10. A Lewandowski-Kurowicka-Joe (LKJ) prior with a shape parameter of 2 was used for the correlation effect among subject-level parameters and change effects, respectively, to represent a prior believe of the correlation being centered on 0.

3.2.1. Priors independent of model selection

The following priors were defined for all models alike:

$\tau_a \sim Cauchy(0, 10),\ \tau_a \in [0, \infty]$

$\tau_b \sim Cauchy(0, 10),\ \tau_b \in [0, \infty]$

$\tau_{\Delta a} \sim Cauchy(0, 10),\ \tau_{\Delta a} \in [0, \infty]$

$\tau_{\Delta b} \sim Cauchy(0, 10),\ \tau_{\Delta b} \in [0, \infty]$

$\sigma \sim Cauchy(0, 10),\ \sigma \in [0, \infty]$

$\boldsymbol{L_u} \sim LKJcorr(2)$

$\boldsymbol{L_v} \sim LKJcorr(2)$

For the models using 3 parameters (*Ex3* and *Crit*) the following priors were additionally defined:

$\tau_c \sim Cauchy(0, 10),\ \tau_c \in [0, \infty]$

$\tau_{\Delta c} \sim Cauchy(0, 10),\ \tau_{\Delta c} \in [0, \infty]$

3.2.2. Priors specific to Lin

$\mu_a \sim Normal(100.8, 8.5)$

$\mu_b \sim Normal(-2.65, 1.8)$

$\mu_{\Delta a} \sim Normal(0, 10)$

$\mu_{\Delta b} \sim Normal(0, 10)$

3.2.3. Priors specific to Ex2

$\mu_a \sim Normal(102.1, 7.7)$

$\mu_b \sim Normal(-0.032, 0.023)$

$\mu_{\Delta a} \sim Normal(0, 10)$

$\mu_{\Delta b} \sim Normal(0, 10)$

3.2.4. Priors specific to Ex3

$\mu_a \sim Normal(55.2, 102.1)$

$\mu_b \sim Normal(-0.085, 0.222)$

$\mu_c \sim Normal(48.7, 108.4)$

$\mu_{\Delta a} \sim Cauchy(0, 50)$

$\mu_{\Delta b} \sim Cauchy(0, 50)$

$\mu_{\Delta c} \sim Cauchy(0, 50)$

3.2.5. Priors specific to Crit

$\mu_a \sim Normal(21.4, 72.4)$

$\mu_b \sim Normal(-22.1, 48.0)$

$\mu_c \sim Normal(9.2, 127.1)$

$\mu_{\Delta a} \sim Cauchy(0, 50)$

$\mu_{\Delta b} \sim Cauchy(0, 50)$

$\mu_{\Delta c} \sim Cauchy(0, 50)$

References

1. Depaoli S, Winter SD, Visser M. The Importance of Prior Sensitivity Analysis in Bayesian Statistics: Demonstrations Using an Interactive Shiny App. Front Psychol 2020; 11:608045.

2. Carpenter B, Gelman A, Hoffman MD, Lee D, Goodrich B, Betancourt M et al. Stan : A Probabilistic Programming Language. J. Stat. Soft. 2017; 76(1):32.

3. Heyward VH, Gibson AL. Advanced fitness assessment and exercise prescription. Seventh edition. Champaign IL: Human Kinetics; 2014.

4. Haff G, Triplett NT, editors. Essentials of strength training and conditioning. Fourth edition. Champaign IL: Human Kinetics; 2016.

5. Morton RH, Redstone MD, Laing DJ. The Critical Power Concept and Bench Press: Modeling 1RM and Repetitions to Failure. Int J Exerc Sci 2014; 7(2):152–60.
